# What Works in Mindfulness Interventions for Medically Unexplained Symptoms? A Systematic Review

**DOI:** 10.31372/20200501.1082

**Published:** 2020

**Authors:** Ruel Billones, Lukkahatai Nada, Leorey Saligan

**Affiliations:** a National Institute of Nursing Research, NIH, United States; b Johns Hopkins University, School of Nursing, United States; c National Institute of Health, United States

**Keywords:** mindfulness, pain, fatigue, cancer, fibromyalgia, chronic fatigue syndrome, irritable bowel movement syndrome

## Abstract

*Background/Purpose:* Mindfulness-based interventions (MBIs) have been used in medically unexplained symptoms (MUS). This systematic review describes the literature investigating the general effect of MBIs on MUS and identifies the effects of specific MBIs on specific MUS conditions. *Methods:* The Preferred Reporting Items for Systematic Reviews and Meta-Analysis Guidelines (PRISMA) and the modified Oxford Quality Scoring System (Jadad score) were applied to the review, yielding an initial 1,556 articles. The search engines included PubMed, ScienceDirect, Web of Science, Scopus, EMBASE, and PsychINFO using the search terms: mindfulness, or mediations, or mindful or MBCT or MBSR and medically unexplained symptoms or MUS or Fibromyalgia or FMS. A total of 24 articles were included in the final systematic review. *Results/Conclusions:* MBIs showed large effects on: symptom severity (*d*  = 0.82), pain intensity (*d*  = 0.79), depression (*d*  = 0.62), and anxiety (*d*  = 0.67). A manualized MBI that applies the four fundamental elements present in all types of interventions were critical to efficacy. These elements were psycho-education sessions specific to better understand the medical symptoms, the practice of awareness, the nonjudgmental observance of the experience in the moment, and the compassion to ones’ self. The effectiveness of different mindfulness interventions necessitates giving attention to improve the gaps that were identified related to home-based practice monitoring, competency training of mindfulness teachers, and sound psychometric properties to measure the mindfulness practice.

## Introduction

Medically unexplained symptoms (MUS) are subjective symptoms that last for more than 3 months and cause a loss of function with little or absent pathology. Patients with MUS spend $256 billion a year on direct health care in the United States (Barsky, Orav, & Bates, 2005; Burton, McGorm, Richardson, Weller, & Sharpe, 2012) and visit both primary and secondary care centers nearly twice as often as patients without MUS (Barsky et al., 2005; Burton et al., 2012; Page & Wessely, 2003).

MUS have complex predisposing (e.g., genetics, experience, and personality trait), precipitating (e.g., life event, stressors, virus), and perpetuating (e.g., sensitization and the hypothalamus pituitary adrenal axis [HPA axis], cognitive and behavioral inhibitor, attention, belief, and response to illness) factors (Deary, Chalder, & Sharpe, 2007). Current management for MUS includes antidepressants and nonpharmacological interventions such as cognitive behavioral therapy (CBT) and mindfulness-based interventions (MBIs), which show small to moderate effectiveness (Bellato et al., 2013; Herwig, Kaffenberger, Jäncke, & Brühl, 2010; Hofmann, Sawyer, Witt & Oh, 2010).

Mindfulness, which has its origin in Buddhism from Asia (Rhys Davids, 1891), is defined as the quality of awareness or consciousness that emerges from intentionally attending to a nonjudgmental and accepting present moment experience, trust, starting with a beginner’s mind, gratitude, and self-compassion (Gu, Strauss, Bond, & Cavanagh, 2015). Mindfulness is a coherent phenomenological description of the nature of the mind, emotion, suffering and its release based on highly refined practices aimed at systematically training various aspects of the mind and the heart. This practice includes the cultivation of compassionate quality of attending to one’s presence and awareness. It is achieved through the simplicity of attending to one’s breath, expanding the awareness to one’s thoughts, emotions, and parts of the body that may carry different sensations (Kabat-Zinn, 2003).

Table 1 shows the similarities and differences of different MBIs. There are two types of MBIs that are commonly used: Mindfulness Based Stress Reduction (MBSR) and Mindfulness Based Cognitive Therapy (MBCT). Unlike traditional CBT that focuses on encouraging patients to maintain or increase pleasant activities, MBIs like MBSR and MBCT focus on awareness and acceptance of the present situation. The assumption of MBI is that people can effectively cope with life stressors if they focus on the present without worrying about the past or the future (Hofmann et al., 2010). MBSR was the first among the two most extensively tested MBIs to be applied in the medical field, followed by MBCT (Segal, Williams, & Teasdale, 2013). MBSR was developed as an education and training vehicle for people with chronic health problems who were also suffering from psychological and emotional stress (Kabat-Zinn, 2013). Interest in MBSR efficacy has grown exponentially from more than 100 published papers in 2005 to more than 1,500 in 2013 and continues to increase up to the present time (Kabat-Zinn, 2013). The application of MBI as an intervention for chronic conditions essentially should have two components for it to be effective: formalized training of the teacher in the MBSR or MBCT curricula and adherence to the manualized programs of both the teacher and the participants (Crane et al., 2017).

**Table 1:** Summary of Mindfulness Based Interventions

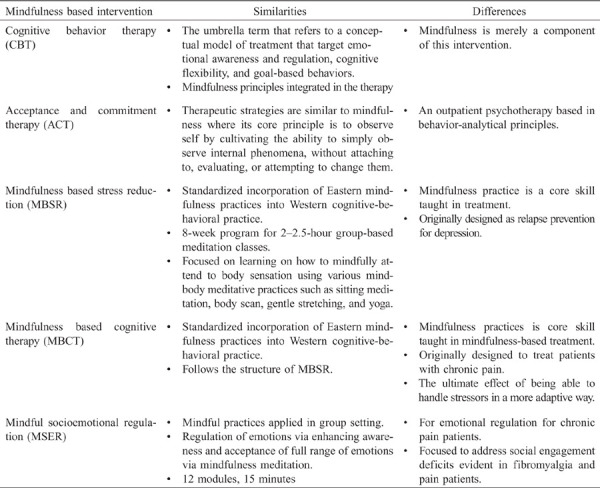

MBIs decrease physical distress from symptoms by balancing sympathetic and parasympathetic responses with meditation exercises by focusing on the breath (Hofmann et al., 2010; Paulson, Davidson, Jha, & Kabat-Zinn, 2013). Previous studies showed promising effectiveness of MBCT on physical symptoms (Bohlmeijer, Prenger, Taal, & Cuijpers, 2010; Skovbjerg, Hauge, Rasmussen, Winkel, & Elberling, 2012; van Ravesteijn, Lucassen, Bor, van Weel, & Speckens, 2013). However, some studies showed no significant difference between patients who received usual care compared to patients who received MBCT, with only up to two thirds of patients who received MBCT showing 50% improvement in their symptoms (Bohlmeijer et al., 2010; Skovbjerg et al., 2012).

Several meta-analyses and systematic reviews investigated the effectiveness of MBIs on vascular disease (Abbott et al., 2014) and cancer (Matchim, Armer, & Stewart, 2011; Shennan, Payne, & Fenlon, 2011). All of these studies reported similar results that MBIs improve mental health, physical symptoms (pain, fatigue, sleep), and psychological symptoms (depression, anxiety). A systematic review on the effectiveness of MBI in improving symptoms of FMS, CFS, IBS, and non-specified/mixed somatization disorder reported an overall small to moderate effect size of MBIs on reducing pain, symptoms severity, depression and anxiety, and improving overall quality of life among patients with these somatization disorders compared to controls (Lakhan & Schofield, 2013). However, these reviews combined the effect of both MBCT and MBSR. A comparative evaluation of the individual effect of MBCT, MBSR, and specific MBI interventions on MUS and its symptoms is needed. The purpose of this review is to examine the individual effectiveness of MBCT, MBSR, and other MBIs in reducing symptoms and improving health outcomes reported by patients with MUS.

## Methods

**Figure 1.**
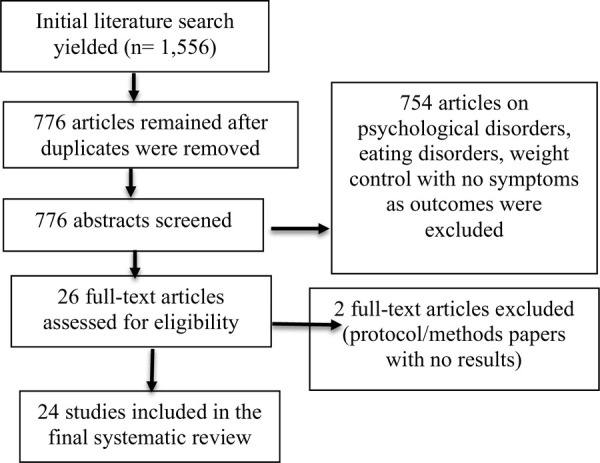
Flow of literature search using PRISMA.

This systematic review was performed according to the Preferred Reporting Items for Systematic Reviews and Meta-Analysis (PRISMA) guidelines (Moher, Liberati, Tetzlaff, Altman, & The PRISMA Group, 2009). The PRISMA guidelines are particularly important for this review to evaluate findings from previous randomized trials involving MBIs. Figure 1 describes the flow diagram used in this review using the PRISMA guidelines. To provide an overall assessment of the quality of the selected clinical trials, the Oxford quality scoring system (Jadad score) was used (Khan, Daya, & Jadad, 1996) to independently assess the methodological quality of these clinical trials. The Jadad score emphasizes specific parts of a study, including randomization, blinding, withdrawal, and dropouts. It is an 11-item assessment the reviewer uses to evaluate the quality of a study based on the description of the study and its methodology. Each item is rated either 0  = does not describe or 1  = describes. Two extra points can be added if the methods of randomization and a double blind are described. Jadad scoring was used in this review because it has the advantage to provide an overview of the quality of randomized clinical trial-related literature. It has the simplicity of its assessment questions and the ease as an assessment performance (Chung, Kang, Jo, & Lee, 2012). Jadad scoring has some limitations, as do other evaluation methods including the Delphi consensus method, the quality criteria from the Cochrane Back Review Group, or even the revised version of the Consolidated Standards of Reporting Trials (CONSORT) statement (Berger & Alperson, 2009).

### Eligibility Criteria

Studies were selected based on the following eligibility criteria: (1) types of study designs: intervention study, randomized controlled trial (RCT), and nonrandomized controlled trial (nRCT); (2) type of participants: studies of patients diagnosed with MUS (including fibromyalgia, chronic fatigue syndrome, irritable bowel syndromFige), regardless of age, condition’s duration, or intensity; (3) studies that enrolled patients diagnosed with an eating disorder, obesity, and psychological disorders were excluded; (4) types of interventions: studies that compared MBIs (both MBCT and MBSR, and other therapies that incorporated mindfulness practice such as CBT, ACT (Acceptance and Commitment Therapy), and MBER (Mindfulness-Based Emotional Regulation)) with either no-treatment, usual care, or any active treatment; (5) types of outcomes: studies that assessed at least one physical symptom, for example, pain, fatigue, depression; (6) length of follow-up: no restrictions regarding length of follow-up were applied; and (7) accessibility of data: only studies that were published full text in English were included.

### Literature Search Strategy

Search engines used included PubMed, ScienceDirect, Web of Science, Scopus, EMBASE, and PsycINFO using the search term for mindfulness, or meditation, or mindful, or MBT, or MBCT or MBSR AND medically unexplained physical symptoms or MUPS or medically unexplained symptoms or MUS or Fibromyalgia or FMS or Irritable bowel syndrome or IBS or Chronic fatigue syndrome or CFS or Somatic disorders.

### Study Selection

After duplicate articles were removed, two reviewers who are co-authors of this review (RB and NL) screened the abstracts of the remaining papers individually and went on to obtain the full papers to carefully screen for eligibility. The studies were then read in detail, with eligible papers included in the final systematic review.

### Study Quality Evaluation

Data extraction was undertaken by one author (RB) and reviewed by another author (NL). When there were disagreements they were resolved by discussion or a vote by the senior author (LS). For each reviewed article, the following data were extracted and included in the assessment of the study’s quality: first author; year of publication; country and research affiliation; population characteristics, including type of sample, age, sex (% female), and number of participants per condition; disease, list of measures used, masking, follow up, comparison with other interventions; type of intervention (e.g., MBSR, MBCT, CBT, ACT); duration of session in weeks; intervention components; background of teacher/therapist; assessment times; method of delivery of sessions (e.g., group, individual, website); nature of the way the study outcomes were assessed; and outcome measures for general medical conditions, quality of life, pain, depression, anxiety, and mindfulness. The first two reviewers (RB and NL) independently reviewed and evaluated the quality of the reviewed articles using Jadad scoring, which is a maximum of 14 points. The higher the score, the better the quality of the study (Khan et al., 1996). The following criteria were applied to generate the Jadad score: (1) objectives of the study defined; (2) outcome measures defined; (3) clear description of inclusion and exclusion criteria; (4) sample size justified; (5) reported as randomized; (6) clear description of interventions; (7) presence of a control group; (8) method to assess adverse effect described; (9) statistical analysis described; (10) identified the study as RCT; (11) appropriateness of randomization; (12) double blinding was reported; (13) double blinding was appropriate; and (14) study withdrawals were reported.

## Results

### Description of Reviewed Articles

Of the 24 articles, 75% were published between 2014 and 2017 and 25% between 2003 and 2009. The majority of the reviewed articles (54%) were authored by groups from North America (USA and Canada), 37% from Europe (United Kingdom, Sweden, the Netherlands, Spain, Switzerland and Germany), and 8% from Asia and the Middle East. The authors’ affiliations and disciplines were from psychology and psychiatry (58%), medicine (primary care, somatic medicine, internal medicine, otolaryngology, alternative medicine) (42%), integrative, physiotherapy and rehabilitation medicine (21%), oncology (5%), neuroscience (5%), social work (5%), and nursing (5%).

### Population and Sample Characteristics

The total number of subjects enrolled in the reviewed articles was 2,126 participants, of which 459 had MUS and 1,667 were controls. The large majority of subjects were females (97.93%). Only three studies included male participants (Berrill, Sadlier, Hood, & Green, 2014; Rimes & Wingrove, 2011; van Ravesteijn et al., 2013). All participants were adults (average age in years  = 42; Europe  = 45, North America  = 49, Asia/Middle East  = 32). The total sample size ranged from 22 subjects in a pilot study (Curtis, Osadchuk, & Katz, 2011) to 342 in a large-scale clinical trial (Cherkin et al., 2016). Of the studies reviewed, eight enrolled fibromyalgia patients, eight enrolled irritable and inflammatory bowel syndrome subjects, and five studies enrolled participants with chronic back pain (*n*  = 342), one Gulf War illness (*n*  = 55), one PTSD (*n*  = 35), and one unclassified MUS (*n*  = 87).

### Methodological Quality

Modified Jadad score for the reviewed articles ranged from 4 to 11, out of a possible 14 points. Four studies had Jadad scores between 11 and 13 (Astin et al., 2003; Cherkin et al., 2016; van Ravesteijn et al., 2013; Zomorodi, Abdi, & Tabatabaee, 2014). The rest of the studies scored ≤10. Larger sample size was the main feature of the studies with higher Jadad scores. Of the 24 studies, 19 were randomized controlled trials, while the rest were case-controlled clinical studies. Five studies used blinding strategies, where the research staff were unaware if the participants of the interventions belonged to the experimental or control groups. Double blinding was not possible in all studies, because of the nature of the interventions used. Most of the reviewed articles (87%) had clearly stated objectives. Several articles had clearly defined outcome measures such as depression/anxiety (*n*  = 9), pain (*n*  = 12), mindfulness skills and attitude (*n*  = 4), and general health status (*n*  = 15). Only one study identified anger as an outcome measure (Amutio, Franco, Pérez-Fuentes Mde, Gázquez, & Mercader, 2015).

Of the 24 studies, 4 assessed study outcomes by clinicians (Ljótsson, Falk et al., 2010; Rimes & Wingrove, 2011; van Ravesteijn et al., 2013; Zomorodi et al., 2014), and the rest using self-report instruments (Amutio et al., 2015; Astin et al., 2003; Banth & Ardebil, 2015; Berrill et al., 2014; Braden et al., 2016; Cash et al., 2015; Curtis et al., 2011; Davis & Zautra, 2013; Garland et al., 2012; Gaylord et al., 2011; Grossman, Tiefenthaler-Gilmer, Raysz, & Kesper, 2007; Kearney, McDermott, Martinez, & Simpson, 2011; Ljótsson, Andréewitch et al., 2010; Schmidt et al., 2011; Sephton et al., 2007; Zernicke et al., 2013). Two studies clearly described the method used to assess adverse effects of the intervention (Banth & Ardebil, 2015; Cherkin et al., 2016). About 75% of the reviewed articles clearly defined the inclusion and exclusion criteria of their studies. Ten of the reviewed articles presented power analyses to demonstrate they had an adequate sample size (Astin et al., 2003; Berrill et al., 2014; Cash et al., 2015; Cherkin et al., 2016; Davis & Zautra, 2013; Kearney et al., 2011; Ljótsson, Andréewitch et al., 2010; Rimes & Wingrove, 2011; Schmidt et al., 2011; van Ravesteijn et al., 2013).

### Effect Size

In general, there was middle to large effect sizes with a 95% confidence interval of MBIs when compared between groups in different time periods on reported symptom severity for patients with fibromyalgia (*d*  = 0.82) (Grossman et al., 2007), post-traumatic stress disorder (*d*  = 0.55) (Kearney, 2012), irritable bowel syndrome (*d*  = 0.83) (Ljotsson, 2011), (*d*  = 0.5) (Zernicke, 2013), (*d*  = 0.05) for pain in gulf war illness (Kearney et al., 2016), in irritable bowel syndrome (*d*  = 0.64) (Ljotsoon, 2010b), (*d*  = 0.78) (Ljotsoon, 2011), (*d*  = 0.62) depression (Kearney et al., 2011), (*d*  = 0.86) Ljotssoon, 2011), and (*d*  = 0.67) anxiety (Grossman et al., 2007). Effect sizes were not calculated, instead, only articles that reported effect sizes were reflected in this paper. See Table 2 for summary of effect size.

**Table 2:** Summary of Effect Size

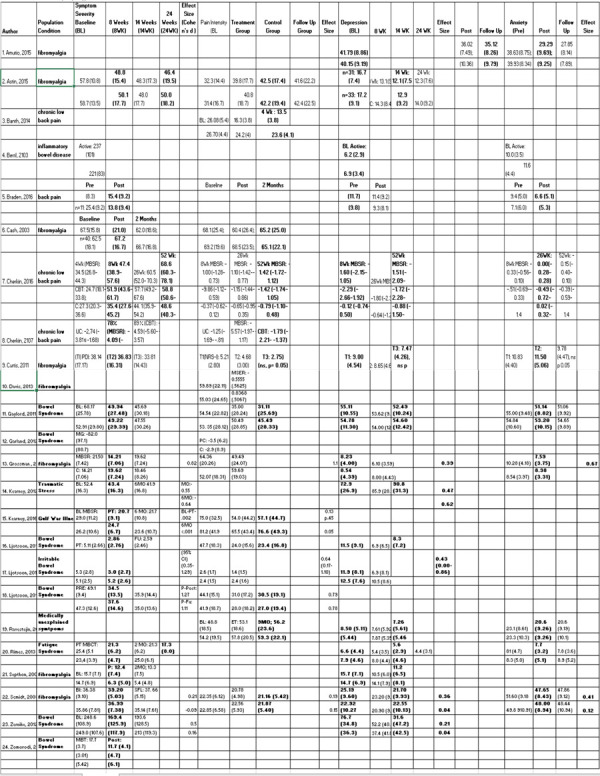

### Intervention Characteristics

Of the 24 articles, nine used MBSR, four used MBCT, two CBT, one Acceptance and Commitment Therapy, one Mindful Socioemotional Regulation, and the rest used a modified MBI to fit the needs of the intended clinical population and the study objectives. One study compared MBSR to CBT (Zomorodi et al., 2014). Table 1 describes specific elements of these MBIs and how they are similar or different from each other.

Standard MBI include mindful movement, beginning the awareness practice with mindful breathing to the different bodily sensations, then observation of one’s thought processes, and the practice of compassion as an attitude to life (Kabat-Zinn, 2013). The modifications made by the authors of the reviewed studies included: (1) duration of the MBIs from 8 to 10 sessions (*n*  = 12, Amutio et al., 2015; Astin et al., 2003; Berrill et al., 2014; Cash et al., 2014; Davis & Zautra, 2013; Grossman et al., 2007; Sephton et al., 2007), (2) introduced with a day of retreat from 4 to 8 hours (*n*  = 3, Kearney et al., 2011; Schmidt et al., 2011; Sephton et al., 2007; Zernicke et al., 2013), (3) shortened the mindful movements (*n*  = 2, Berrill et al., 2014; Braden et al., 2016), (4) provided supplemental psychoeducational modules (*n*  = 3, Astin et al., 2003; Davis & Zautra, 2013; Grossman et al., 2007), (5) manualized format of sessions and added homework activities (*n*  = 5, Amutio et al., 2015; Berrill et al., 2014; Davis & Zautra, 2013; Sephton et al., 2007), (6) used consistent monitoring and follow up of home practice (*n*  = 5, Amutio et al., 2015; Berrill et al., 2014; Davis & Zautra, 2013; Sephton et al., 2007), (7) had classes lasting from 90 minutes to 2.5 hours per session (*n*  = 4,

Astin et al., 2003; Cash et al., 2014; Kearney et al., 2011; Sephton et al., 2007), and (8) used group-based sessions (*n*  = 19, Amutio et al., 2015; Astin et al., 2003; Banth & Ardebil, 2015; Berrill et al., 2014; Braden et al., 2016; Cash et al., 2014; Curtis et al., 2011; Davis & Zautra, 2013; Garland et al., 2012; Gaylord et al., 2011; Grossman et al., 2007; Kearney et al., 2011; Ljótsson, Andréewitch et al., 2010; Schmidt et al., 2011; Sephton et al., 2007; van Ravesteijn et al., 2013; Zernicke et al., 2013; Zomorodi et al., 2014). Two studies were conducted purely online and thus lacked the above characteristics (Davis & Zautra, 2013; Ljótsson, Falk et al., 2010).

In addition to the above, Table 3 summarizes the elements of the MBI curricula. Ten of the reviewed studies identified the manualized format of the intervention to include recording the daily practice of MBIs and applying lessons learned from the didactic sessions (Amutio et al., 2015; Berrill et al., 2014; Garland et al., 2012; Gaylord et al., 2011; Kearney et al., 2011; Ljótsson, Andréewitch et al., 2010; Rimes & Wingrove, 2011; Sephton et al., 2007; Zernicke et al., 2013). Only one study established a system to follow up and capture home practice sessions, where the follow up was done on a one-on-one basis versus doing the follow up in groups (Ljótsson, Falk et al., 2010). This is important because it is in the home practice session where the consistency is established, which is the key to the efficacy of the intervention (Crane et al., 2017).

**Table 3:** Elements of Mindfulness Based Intervention Curriculum

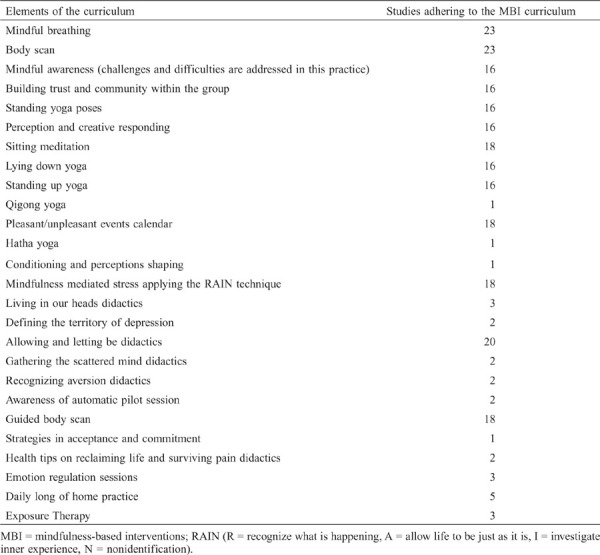

Guidance was provided during the MBIs. Guidance consisted of group coaching (*n*  = 4), feedback on assignments (*n*  = 8), and didactics on mindfulness attitudes (*n*  = 16). The training and credentials of MBI providers varied, to include MBSR, MBCT, or multi-convergent therapy (MCT) trained and/or certified instructors/therapists (*n*  = 15), graduate psychology students (*n*  = 2), licensed social workers (*n*  = 1), licensed psychologists (*n*  = 1), psychiatrists trained in CBT (*n*  = 1), and a certified yoga instructor (*n*  = 1). The length of experience of the MBI providers ranged from 2 to 29 years (Amutio et al., 2015; Berrill et al., 2014; Garland et al., 2012; Gaylord et al., 2011; Kearney et al., 2011; Ljótsson, Andréewitch et al., 2010; Rimes & Wingrove, 2011; Sephton et al., 2007; Zernicke et al., 2013).

### Adherence to the Intervention

Adherence to the interventions are completed interventions of at least 8 sessions, ?4 sessions up to 10 weeks. Of the 24 studies, 16 had intervention adherence. The other eight, did not specify if the frequency to attend the sessions was less than 4 times, or they simply did not mention the consistency of the participations during the whole duration of the intervention.

## Discussion

### Main Findings and Its Clinical Relevance

The review showed that MBI have a middle to large effect size with a 95% confidence interval of MBIs when compared between groups in different time periods on reported symptom severity for patients with fibromyalgia (*d*  = 0.82) (Grossman, 2007), post-traumatic stress disorder (*d*  = 0.55) (Kearney, 2012), irritable bowel syndrome (*d*  = 0.83) (Ljotsoon, 2011), (*d*  = 0.5) (Zernicke, 2013), (*d*  = 0.05) for pain in gulf war illness (Kearney et al., 2016), in irritable bowel syndrome (*d*  = 0.64) (Ljotsoon, 2010b), (*d*  = 0.78) (Ljotsoon, 2011), 2011, (*d*  = 0.62) depression (Kearney et al., 2011), (*d*  = 0.86) Ljotssoon, 2011), and (*d*  = 0.67) anxiety (Grossman et al., 2007) (See Table 2). Findings from this review provided new evidence that these forms of interventions have the potential to manage complex conditions such as MUS and identified specific MBI modalities that were effective in reducing particular symptoms of a specific MUS.

The 75% increase of MBI studies between 2014 and 2017 suggests renewed interest in these interventions and the growing evidence of their efficacy in MUS. This increase could be brought about by the increase in incidence of MUS diagnoses, and yet the therapeutic options have been limited (van Ravesteijn et al., 2013). The receptivity in the West to alternative medicine, complemented by the establishment of different institutions such as the National Center for Complementary and Integrative Health in the National Institutes of Health (NIH) embracing these forms of eastern interventions, have cultivated an open reception to such modalities (Barnett & Shale, 2012).

The increased interest in the West to MBIs is reflected in the authorship of reviewed articles, where most were authored by U.S. and European research teams. Considering that the historical core of MBIs is anchored in eastern healing, there may be a discrepancy in how the effect of MBIs is perceived or assessed. The interventions are a way of being and not a set of lists of activities to be accomplished during the day. This dynamic of subjectivity and the stance of “being” rather than of “doing,” pose a challenge to studies conducted in the West. In the eastern practice of MBIs, each session is adapted based on the relational/emotional dynamics between the teacher and participant at that particular time (Dimidjian & Segal, 2015). This dynamic relationship was a challenge to capture and this was not mentioned in any of the reviewed articles. The study outcomes of the reviewed articles used static forms.

The competency background of the teachers in the reviewed articles also varied. The academic formation of a teacher with a strong background in the behavioral sciences is necessary to facilitate the experiences that come out from the mindfulness sessions. The personal practice of mindfulness for the teacher is critical (Dimidjian & Segal, 2015). The teacher’s self-awareness brought about by the mindfulness-based practices becomes foundational to assist in guiding the participants who begin their practices. The competency of teachers must be standardized (Dimidjian & Segal, 2015). Currently, MBCT and MBSR go through stages of formalized certification before a practitioner is recognized to be a qualified professional to administer them.

The reviewed articles had disproportionate sampling. Only 459 MUS patients were enrolled, with 1,667 healthy controls. This disparity may have an influence on the effect sizes reported. One article had a high drop-out rate (61% adherence) (Cherkin et al., 2016), and three studies had no control groups (Astin et al., 2003; Rimes & Wingrove, 2011; van Ravesteijn et al., 2013).

### Gaps in Research

Several gaps in knowledge and practice were identified in the review that can be helpful for clinicians and researchers. First, the samples used in the reviewed articles were heterogenous. Some participants had prior exposure or experience in doing mindfulness practice as a formalized course before participating in the study being mixed with others whose participants had no prior experience (Amutio et al., 2015). There were also varying history and length of time that the participants had had the medical conditions (Astin et al., 2003; Cash et al., 2015; Davis & Zautra, 2013). Second, the review revealed that certification and training of teachers administering MBIs need to be revisited by professional organizations. Current standards according to the American Psychological Association are conforming to the six-element guidelines that MBCT and MBSR certification bodies published as competency requirements. Third, there seems to be a lack of standardization in the implementation of MBIs. Only two of the reviewed articles incorporated the necessary elements for an effective mindfulness practice (Kearney et al., 2011; van Ravesteijn et al., 2013; see Table 4). Third, measures used in the reviewed articles were static tools whose theoretical constructs were based on certain psychometric assumptions, while MBIs operate in a dynamic, relationship-based paradigm. Innovative approaches to capture this dynamic paradigm will be crucial to determine the actual effect of MBIs on symptoms and various conditions. Lastly, the reviewed articles used MBIs that incorporated topics driven by known medical profiling of specific conditions without considering the mindfulness theoretical construct driven solely by the patient’s experience as the main framework of the intervention.

**Table 4:** Adherence to the Critical Elements of the MBI Curriculum (Ameli, 2014; Dimidjian & Segal, 2015)

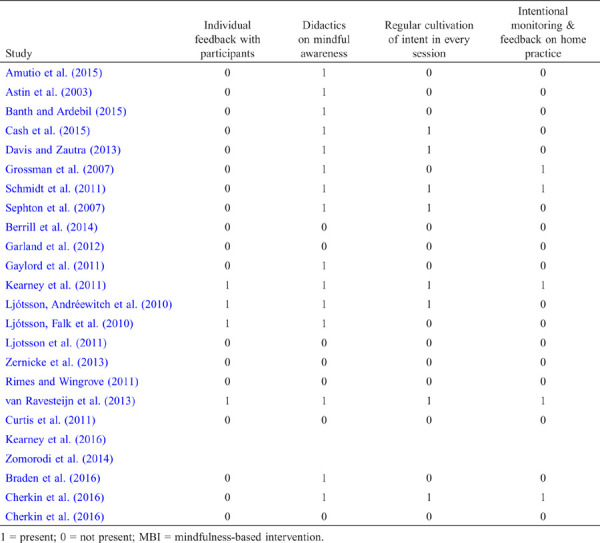

### Recommendations for Future Research

Moving forward, future studies should focus on standardizing these MBIs to be able to identify specific elements of the mindfulness practice that bring out the mechanism of behavior change. The following recommendations can further advance the science of MBI: (1) the adaptation of the mindful movement as a core component to these MBIs because physical exercises are tailor-made for specific medical conditions; (2) the provision of a qualified supervisor to oversee MBI practitioners to ensure faithful adherence to the mindfulness theoretical fundamentals. This could be done by supervisors getting certified in conducting MBSR and MBCT interventions; (3) implement the “buddy system,” so patients or a group of individuals who share similar conditions can walk alongside during the course of the study; and (4) in addition, the review showed lack of consistency in home practices that impacted the outcomes of the study. There is a need to do longitudinal studies and long-term follow up, cultivating the incorporation of mindfulness lifestyles.

Faithful adherence of teachers to the MBI curricula both in content of the program and in the process of implementing the program, the validation of manualized MBI applied to the specific medical condition are elements that support in capturing the dynamic patient experience while doing mindfulness practice. Evaluations will be based on these elements as they impact the symptom experience.

## Conclusion

The review confirmed that MBIs such as MBSR, MBCT, and others (CBT, MBER, MCT, ET) have beneficial effects on MUS and the quality of health outcomes. To identify the behavioral mechanisms that bring about the efficacy of MBI elements is an important gap that should be the focus of future studies. These elements are: the momentary awareness of experience in the cultivation of mindful breathing exercises, the didactics on mindful awareness tailored to the specific medical conditions of the participants, mindful exercises, awareness of one’s intention to practice mindfulness, the role of group members’ feedback, the consistency of the home practice, and the exercise of self-compassion. This review provided an evidence of the efficacy of MBIs on different MUS, as well as identified the gaps in research methodologies that will be helpful for nurses and other health care professionals, in order to deliver effective MBIs in different medically undiagnosed conditions. The findings of this review are particularly informative for the Asian/Pacific Islander population who have historically been linked to increase use of complementary and alternative therapies such as MBIs (Nadin et al., 2007).

## Declaration of Conflicting Interests

The authors have no conflicts of interest to declare.
